# New Hosts for Equine Herpesvirus 9

**DOI:** 10.3201/eid1410.080703

**Published:** 2008-10

**Authors:** Mark D. Schrenzel, Tammy A. Tucker, Taryn A. Donovan, Martin D.M. Busch, Annabel G. Wise, Roger K. Maes, Matti Kiupel

**Affiliations:** Zoological Society of San Diego, Escondido, California, USA (M.D. Schrenzel, T.A. Tucker); Animal Medical Center, New York, New York, USA (T.A. Donovan); IDEXX Laboratories, Ludwigsburg, Germany (M.D.M. Busch); Michigan State University, Lansing, Michigan, USA (A.G. Wise, R.K. Maes, M. Kiupel)

**Keywords:** equine herpesvirus-9, polar bear, Grevy’s zebra, Persian onager, inter-species, transmission, dispatch

## Abstract

Equine herpesvirus 9 was detected in a polar bear with progressive encephalitis; the source was traced to 2 members of a potential equid reservoir species, Grevy’s zebras. The virus was also found in an aborted Persian onager. Thus, the natural host range is extended to 6 species in 3 mammalian orders.

Equine herpesvirus (EHV) 9, a varicellovirus in the subfamily *Alphaherpesvirinae*, is the newest member of the equine herpesviruses. EHV-9 is most closely related to the recently emergent neurotropic pathogen, EHV-1, but was first described in an outbreak of disease in Thomson’s gazelles (*Gazella thomsoni*) and subsequently in a giraffe (*Giraffa camelopardalis* *reticulata*) with encephalitis (*1*–*3*; M. Kiupel, pers. comm.). Initial findings of the virus’ virulence and potential for transmission between equids and artiodactyls were alarming and provided the impetus for experimental studies, which showed that disease could be induced in members of an additional 8 mammalian taxa: dogs, cats, horses, mice, hamsters, pigs, goats, and marmosets (*4*–*6*).

Preliminary data suggest that equids are natural hosts of EHV-9 and experience little or no illness when infected. Seroconversion was detected in 60% of wild Burchell’s zebras (*Equus* *burchelli*) in Tanzania without any associated illness (*3*). When domestic horses (*E. caballus*) were infected by intranasal inoculation with 10^7^ PFU of EHV-9, only mild disease developed and clinical signs were limited to transient fever (*6*). Natural coadaptation of EHV-9 and equids was corroborated by the severity of disease seen in nonequids: fulminant encephalitis with extensive neuronal necrosis in both spontaneous cases and experimental models (*2*–*6*). Antigenic and genetic similarities of EHV-9 with other equine herpesviruses are, likewise, consistent with the theory that equids are primary hosts. However, active infection has never been conclusively documented in any member of the Equidae (*2*–*6*).

The potential vulnerability of diverse species to EHV-9 has raised concern about the virus as an anthropozoonotic pathogen (*2*–*6*). Of additional note is the lack of known reservoir species and infectivity in nonlaboratory environments. We report natural EHV-9 infection and resultant disease in an ursid and 2 equid species, confirming the virus’ promiscuity and pathogenicity and supporting its natural residence in wild equids.

## The Study

**Figure 1:**
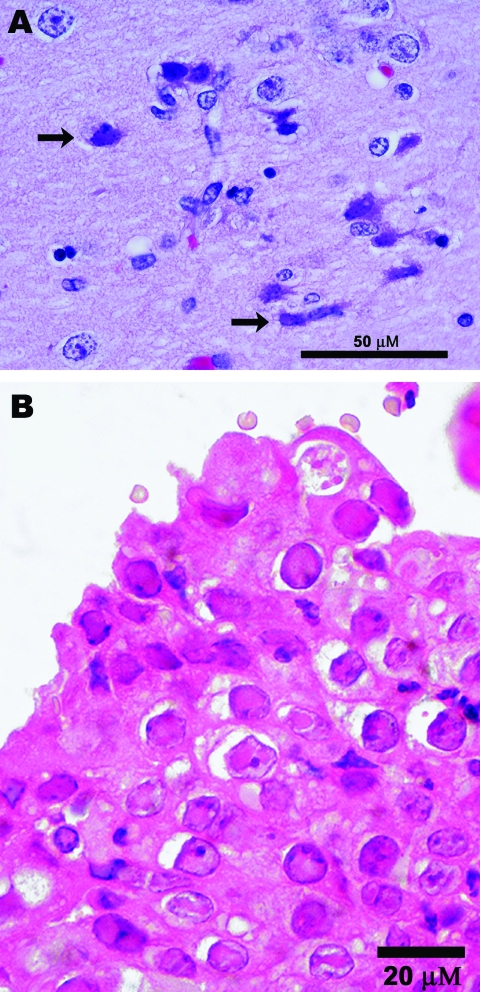
Photomicrographs showing A) encephalitis with neuronal necrosis and intranuclear inclusions (arrows) in a polar bear (*Ursus maritimus*); scale bar = 50 μm; hematoxylin and eosin stain; and B) Grevy’s zebra (*Equus grevysi*) with acute rhinitis with eosinophilic inclusions in respiratory epithelium; scale bar = 20 μm; hematoxylin and eosin stain.

In July, 2007, a 12-year-old polar bear (*Ursus* *maritimus*) in a zoological garden in San Diego, California, showed progressive neurologic signs that were refractory to therapy. The animal was housed ≈200 feet from a herd of recently relocated Grevy’s zebras (*E. grevysi*). Ultimately, the bear was euthanized. Postmortem examination showed nonsuppurative meningoencephalitis with neuronal and glial intranuclear inclusion bodies (Figure 1, panel **A**). PCR targeting of conserved regions of herpesvirus DNA polymerase genes identified a virus in the brain with 98% homology to EHV-1 in a 165-bp segment of DNA (*2*,*7*). Subsequently, 2 other PCRs targeting an additional 742 bp of the DNA polymerase gene (sense primer 5′-GCATYWTCCCCCCGTTKATRAC-3′ and antisense primer 5′-ATAGYSAARRCCACGCCTTY-3′) and 1,181 bp of the glycoprotein B (gB) gene (sense primer 5′-CTTGTGAGATCTAACCGCAC-3′ and antisense primer 5′-GGGTATAGAGCTTTCATGGGG-3′) identified the virus as EHV-9 and enabled more precise strain determination (*2*). DNA segments of the terminase and gB genes were also characterized to generate additional phylograms and to compare with molecular findings from other animals in this study (*8*,*9*).

Before the polar bear case, EHV-9 had been detected at the same zoological garden in 2 Grevy’s zebras from the same herd, which had been relocated near the polar bears. One of the infected Grevy’s zebras was 8 days old and had viral interstitial pneumonia; the other was an adult with rhinitis and intranuclear inclusion bodies (Figure 1, panel **B**). Both zebras were immunocompromised as a result of other concurrent conditions (i.e., sepsis, diarrhea, and tracheitis in the neonate and a traumatic nonhealing wound with fungal infection in the adult). EHV-9 was also found by a retrospective analysis of tissues from an aborted Persian onager (*E. hemionus onager*) fetus from a zoological park in Washington, DC (*10*). The onager fetus was aborted after the dam came in close proximity to a Grevy’s zebra (*10*). PCR and DNA sequencing analyses of the DNA polymerase showed that the zebras and the onager had an EHV-9 strain identical to that found in the polar bear. PCR results for other potential pathogens (e.g., EHV-1, adenovirus, chlamydiae, rickettsiae, rabies, paramyxovirus, and West Nile virus) were negative in the polar bear, zebras, and onager.

**Figure 2:**
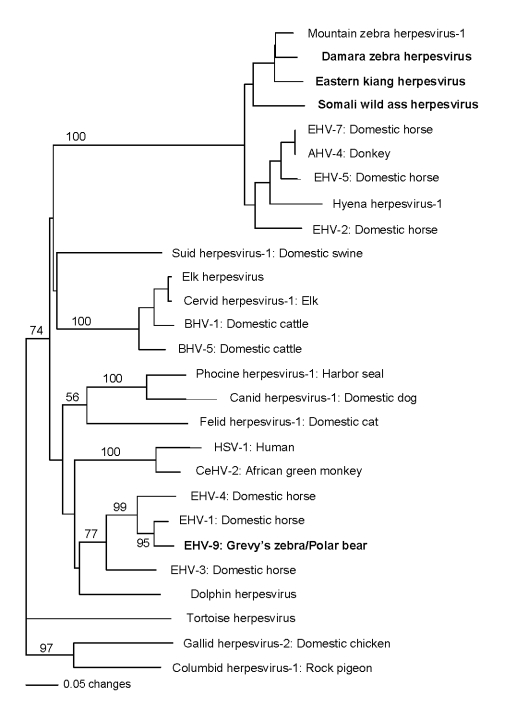
Phylogram of all equine herpesviruses and related viruses from other animals and their respective hosts created from a predicted amino acid segment of the DNA polymerase gene. All sequences obtained in this study are in **boldface**; bootstrap values >1,000 replicates are denoted. Note clustering of equine gammaherpesviruses and paraphyletic grouping of equine herpesviruses (EHV) -1, EHV-4, and EHV-9 with primate herpesviruses and dolphin herpesvirus. Sequence accessions, sources, and abbreviations are as follows: mountain zebra (*Equus zebra*) herpesvirus AAS75147; Damara’s zebra (*E. burchellii antiquorum*) herpesvirus EU17155; eastern kiang (*E. kiang* *holdereri*) herpesvirus EU17156; Somali wild ass (*E. asinus somalicus*) herpesvirus EU71754; EHV-7 from domestic horse (*E. caballus*) ABW04888; asinine herpesvirus 4 (AHV-4) from donkey (*E. asinus*) AAL14770; hyena (*Crocuta crocuta*) herpesvirus AB121852; EHV-2 from domestic horse ABS81334; suid herpesvirus 1 from domestic swine (*Sus scrofa*) DAA02153; elk (*Cervus canadensis*) herpesvirus ABC58213; cervid herpesvirus 1 from elk (*Cervus canadensis*) ABC58214; bovine herpesvirus 1 (BHV-1) from domestic cattle (*Bos taurus*) ABC58212; bovine herpesvirus 5 (BHV-5) from domestic cattle NP_954917; phocine herpesvirus 1 from Pacific harbor seal (*Phoca vitulina richardsii*); canid herpesvirus 1 from domestic dog (*Canis lupus familiaris*) ACB46532; felid herpesvirus 1 from domestic cat (*Felis cattus* *domesticus*) CAA12264; human herpesvirus 1 (HSV-1) from humans (*Homo sapiens*) AAL49731; cercopithecine herpesvirus 2 (CeHV-2) from African green monkey (*Cercopithecus aethiops sabaeus*) YP_164473; EHV-4 from domestic horse; EHV-1 from domestic horse BAG24215; EHV-9 from Grevy’s zebra (*E. Grevy’si*), Persian onager (*E. hemionus onager*), and polar bear (*Ursus* *maritimus*) EU17146, EU17147, and EU17149; EHV-3 from domestic horse AF514779; and dolphin herpesvirus from bottlenose dolphin (*Tursiops truncatas*) AAV1097. The following 3 sequences were outgroups: tortoise herpesvirus from land tortoise (*Testudo* *horsfieldii*) ABC70838, gallid herpesvirus 2 from domestic chicken (*Gallus domesticus*) AAC55651, and columbid herpesvirus 1 from rock pigeon (*Columba livia*) ABP93390.

To test the possibility that other reservoirs of EHV-9 exist, we conducted a molecular survey using herpesvirus consensus-based PCRs for DNA polymerase, terminase, and gB gene segments and EHV-9–specific PCRs. Samples were blood and nasal swabs from a Damara’s zebra (*E. burchellii antiquorum*), Somali wild ass (*E. asinus somalicus*), and eastern kiang (*E. kiang* *holdereri*) from the aforementioned San Diego zoological park (*6*–*8*). Novel herpesviruses, but not EHV-9, were detected in all 3 equid species. Distance analyses of DNA and predicted amino acid sequences from these novel herpesviruses, EHV-9, and previously described viruses from a variety of animals were done by using PAUP (*11*). Phylograms from neighbor-joining distance and parsimony methods showed a partitioning of the equid herpesviruses into 2 clades, representing alpha- and gammaherpesviruses. One clade included EHV-9, EHV-1, and EHV-4 and was paraphyletic to herpesviruses from primates; the other clade comprised equid herpesviruses and a hyena herpesvirus that branched separately from other viruses (Figure 2). Phylograms derived from gB and terminase gene segments, individually or concatenated, produced comparable results. GenBank accession numbers for all sequences from this study are EU17146–EU17156.

## Conclusions

This report demonstrates interspecies transmissibility of EHV-9 on an ordinal level and confirms the virus’ neurotropic pathogenicity in nonequids. Our data show that EHV-9 is able to naturally infect and cause encephalitis in polar bears that had no direct contact with an animal point source. Previous reports described EHV-9 infection and encephalitis in Thomson’s gazelles and a reticulated giraffe that directly commingled with zebras (*2*, *12*; M. Kiupel., pers. comm*.*). By contrast, the polar bear from our study was probably infected by a fomite contaminated by an adjacent herd of Grevy’s zebras. Animal-to-animal transmission, although possible, is extremely unlikely because of the space separating the polar bear and zebra enclosures. In either instance (direct animal-to-animal or indirect fomite transmission), the infectivity of EHV-9 for polar bears would have been substantial.

Our data also demonstrate naturally occurring EHV-9 infection in equids and suggest that >1 species of zebra may be hosts. Findings point toward Grevy’s zebras as 1 reservoir species. In addition to finding active infection with EHV-9 in the zebras, the nature of the lesions and association of disease with compromised immunity and a perinatal animal emulated the host–pathogen dynamics of EHV-1 in domestic horses (*13*). A prior serologic survey that used virus-specific neutralization assays suggested that Burchell’s zebras may also be natural hosts of EHV-9, although features of active infection, such as latency and viral shedding, were not investigated in that study (*3*). Excluding experimental studies, neither titers to EHV-9 nor the virus itself have been found in domestic horses (*6*).

An additional finding was the taxonomic affinity of EHV-9 and other equine alphaherpesviruses to herpes simplex virus 1 and cercopithecine herpesvirus 2. EHV-1 is endemic in domestic horses and can cause pneumonia, myeloencephalitis, and abortion (*13*). EHV-9 is highly related to EHV-1 but unique in its ability to cause disease in a variety of other mammals, including primates (*1*–*5*). This ability has drawn attention to the possibility of eventual zoonotic transmission and supports grading of EHV-9 with other incipient semigeneralist pathogens (*4*–*6*).

Redefining the host range of EHV-9 raises new issues regarding the anthropogenic effects of assembling diverse species in zoological gardens and the growing interface between wildlife, domesticated animals, and humans. Through comparative studies with other equine herpesviruses such as EHV-1, EHV-9 can be now considered of preeminent value as a model for understanding how viruses cross species barriers.
